# SNPs in DNA repair or oxidative stress genes and late subcutaneous fibrosis in patients following single shot partial breast irradiation

**DOI:** 10.1186/1756-9966-31-7

**Published:** 2012-01-24

**Authors:** Elisabetta Falvo, Lidia Strigari, Gennaro Citro, Carolina Giordano, Genoveva Boboc, Fabiana Fabretti, Vicente Bruzzaniti, Luca Bellesi, Paola Muti, Giovanni Blandino, Paola Pinnarò

**Affiliations:** 1 Laboratory of Pharmacokinetic/Pharmacogenomic, Regina Elena National Cancer Institute, Rome, Italy; 2 Laboratory of Medical Physics and Expert Systems, Regina Elena National Cancer Institute, Rome, Italy; 3 Department of Radiation Oncology, Regina Elena National Cancer Institute, Rome, Italy; 4 Department of Oncology Juravinski Cancer Center, McMaster University Hamilton, Hamilton, ON, Canada; 5 Department of Public Health, Harvard University, Boston, MA, USA; 6 Translational Oncogenomic, Regina Elena National Cancer Institute, Rome, Italy

**Keywords:** Radiotherapy, Breast cancer, Polymorphisms, Late effects, Fibrosis

## Abstract

**Background:**

The aim of this study was to evaluate the potential association between single nucleotide polymorphisms related response to radiotherapy injury, such as genes related to DNA repair or enzymes involved in anti-oxidative activities. The paper aims to identify marker genes able to predict an increased risk of late toxicity studying our group of patients who underwent a Single Shot 3D-CRT PBI (SSPBI) after BCS (breast conserving surgery).

**Methods:**

A total of 57 breast cancer patients who underwent SSPBI were genotyped for SNPs (single nucleotide polymorphisms) in XRCC1, XRCC3, GST and RAD51 by Pyrosequencing technology. Univariate analysis (ORs and 95% CI) was performed to correlate SNPs with the risk of developing ≥ G2 fibrosis or fat necrosis.

**Results:**

A higher significant risk of developing ≥ G2 fibrosis or fat necrosis in patients with: polymorphic variant *GSTP1 *(Ile105Val) (OR = 2.9; 95%CI, 0.88-10.14, *p *= 0.047).

**Conclusions:**

The presence of some SNPs involved in DNA repair or response to oxidative stress seem to be able to predict late toxicity.

**Trial Registration:**

ClinicalTrials.gov: NCT01316328

## Background

Conservative surgery followed by adjuvant radiotherapy(RT) to whole breast has become widely accepted as a standard of care for women with early breast cancer. In particular, a number of studies [1-4] reported that most (81%-100%) intra breast tumour recurrences after breast conserving surgery (BCS) occur in close proximity to the tumour bed, so providing the rationale of Partial Breast Irradiation (PBI) an adjuvant RT limited to the Index Area i.e. the area of breast only including the primary tumour bed and the surrounding tissue. In addition, the delivery of radiation dose to smaller target volume by PBI is expected to reduce radiation-related toxicity. Thus, the so-called Accelerated Partial Breast Irradiation (APBI), where only the Index Area is irradiated in 1-10 fractions at high dose/fraction, has been promoted in phase I-III trials designed to test feasibility and equivalence with standard Whole Breast Irradiation (WBI) in properly selected low risk early breast cancer patients after BCS [5]. However, a remarkably high rate of late toxicity has been reported by some Authors a few years after follow up with this APBI approach [6,7]. A high late toxicity rate was also observed in our cohort, after single shot of PBI (SSPBI) [8]. Thus the possibility to predict patient outcome based on marker genes correlated with radio-induced toxicity was investigated.

The interaction of RT with living tissue generates, directly or transitorily, reactive oxygen species (*ROS) *triggering a series of inflammation reactions. Adaptation to oxidative stress occurs by activating genes that characterize the cellular responses to this type of stress and generates a series of processes including DNA repair pathways, cell cycle arrest, antioxidant enzymes and secretion of cytokines that are suspected to play a central role in the development of mainly late normal tissue damage [9,10]. These mechanisms, eventually lead to avoiding extensive DNA damage, cell death [11], and inflammatory process, that may enhance *ROS *production, thus, contributing to the formation of fibrogenesis and tissue remodelling [12]. In particular, *Glutathione-S-Transferase *(*GSTs*) are antioxidant enzymes which are classified into the following classes: alpha (*GSTA*), mu (*GSTM*), pi (*GSTP*), theta, sigma, and kappa. Under conditions of stress the *GSTP1 *class is implicated in pro-apoptotic signalling and may mediate cytotoxicity [13-15]. Two independent studies recently carried out on BC patients have reported a significant association between the *GSTP1 *105Val variant (313 G) and an increased risk of developing acute or late adverse reactions induced by radiation therapy [9,16].

In addition, *XRCC1 (X-ray Repair Cross-Complementation group 1), XRCC3 *(*X-ray Repair Cross-Complementation group 3*) *RAD51*, genes involved in the DNA repair process may influence susceptibility to side effects in patients receiving radiation therapy given that DNA is a direct target for ionizing radiation [17-20].

Various studies [21-23] showed a significant association between the polymorphic nature of these genes and the possibility of developing biomarkers or predictive assay for radio-sensitivity in breast cancer patients.

To correlate the genetic variation and association between the development of late effects [24,25], we investigated the following specific polymorphic genes: *XRCC1 *(Arg399Gln), *XRCC3 *(5'UTR and Thr241Met), *GSTP1 *(Ile105Val) and *RAD51*.

## Methods

From March 2006 to January 2008, patients who underwent BCS and a sentinel node biopsy and/or axillary dissection for early breast adenocarcinoma and met eligibility criteria were treated in the prone position with an adjuvant single dose 3D-CRT APBI schedule to the Index Area. The eligibility criteria included being aged ≥ 48 years with a life expectancy of at least 5 years, post-menopausal status, histologically proved cancer, non lobular, adenocarcinoma of the breast, primary tumours ≤ 3 cm, negative surgical margins (≥ 2 mm), negative sentinel nodes or < 4 positive axillary nodes, no extra-capsular extension, no previous radiotherapy. The exclusion criteria included patients with multicentric disease, extended intraductal component (EIC > 25%), Paget's disease of the nipple, lobular adenocarcinoma, and distant metastases.

A dose of 18 (in 4 patients) or 21 Gy (in 60 patients), normalized to the PTV mean dose, was prescribed in a single session. Major technical details of our approach have been previously reported in detail in a distinct paper [26]. Some radiobiological considerations on single dose, time factors, clonogenic cell density and dose constraints are reported in distinct papers [27-30].The study was conducted in accordance with the Helsinki Declaration. Each patient was informed about the study protocol in both verbally and in writing (informed consent) in advance. The patient was given ample opportunity to request relevant information regarding the study and decide on their own whether to participate in the protocol. The protocol was approved by the local Ethics and Scientific Committee of the Regina Elena Italian National Cancer Institute (reference number IFO-84/10). (The trial has been registered at the ClinicalTrials.gov website and it is identified as NCT01316328).

Fibrosis was assessed using the National Cancer Institute's Common Terminology Criteria for Adverse Events (CTCAE, version 3.0) [31]. Fat necrosis was also scored according to the system proposed by Lövely et al. [32]. The end-point of this study is Grade 2 or more fibrosis or fat necrosis. Toxicity was defined as late if it occurred ≥ 6 months after radiotherapy.

All subjects enrolled in the study provided a blood sample, approximately 5 ml, in sterile tubes containing ethylenediaminetetracetic acid (EDTA). Whole blood samples for DNA analyses were immediately frozen at -80°C until processing. Total genomic DNA of samples was extracted from blood leukocytes using the kit QIAmp (DNA blood Mini Kit, Qiagen, Valencia, CA) following the manufacturer's instructions. DNA quality was evaluated by spectrophotometer analysis (NanoDrop instrument). PCR reactions for these polymorphic genes were performed as Real Time PCR using Rotorgene Instrument (Corbett) following PCR (Polymerase Chain Reaction) conditions provided by the manufacturer's instructions. The polymorphic genes: *XRCC3 *C18067T (Thr241Met), *XRCC3 *A4541G (5'-UTR untranslated region), *XRCC1 *G28152A (Arg399Gln), *GSTP1 *A313G (Ile105Val) *RAD51 *G135C (untranslated region including in the commercial kits for Radiotherapy Response) (Diatech company) were evaluated. The polymorphic genes were analyzed using Pyrosequencing technologies (instrument PyroMark MD-Biotage, Uppsala, Sweden) according to a previously published method [33].

The first step of the study was designed to correlate SNPs of genes and acute effects (i.e. erythema) [34]. We assumed an erythema rate of 20% and 54% in patient groups at low and high risk, respectively, (groups were identified based on the absence/presence of the above polymorphisms alone or in combination). Thus the minimum sample size was 56 patients with α = 0.05, 2-tailed test and a power of the study of 80%. More radiosensitive patients are expected to show an increased number of acute, as well as, late effects. Thus, we also decided to investigate in a second step the *late fibrosis/fat necrosis and *the following polymorphisms: *XRCC3 *C18067T (Thr241Met), *XRCC3 *A4541G (5'-UTR), *XRCC1 *G28152A (Arg399Gln), GSTP1 A313G (Ile105Val) and *RAD51 *G135C (untranslated region). Moreover, we also analyzed combined genotypes according to data from literature.

Tests for statistical significance were performed with the chi-square and *t*-test for categorical and continuous variables, respectively. Odds ratios (ORs) and 95% confidence intervals (CIs), Chi-squared and Fisher exact (2-sided) tests were calculated. An OR > 1.0 indicates an increased risk of fibrosis in patients with polymorphic gene. All tests were two-sided and considered to be statistically significant with a *p*-value of *p *= 0.05.

## Results

**Figure 1:**
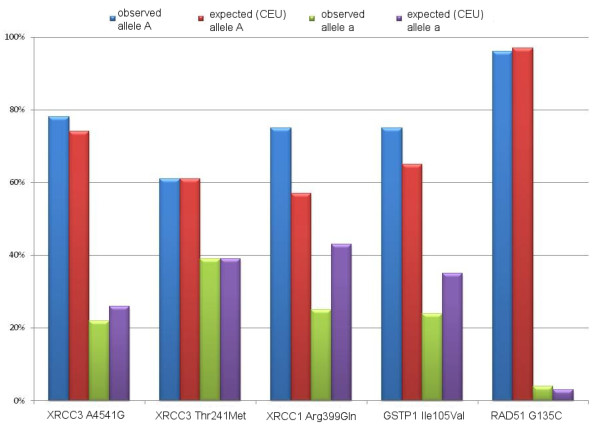
**Polymorphism distribution**.

To these study purposes, i.e. determining polymorphisms predicting late toxicity, we recruited 57 patients treated with SSPBI from March 2006 to January 2008. Out of 57 patients, 15 (26%) were also treated with adjuvant non-concomitant chemotherapy. The adjuvant chemotherapy had been completed 3-4 weeks before RT with the exception of one patient (underwent chemotherapy one-week after SSAPBI). Adjuvant hormonotherapy, as indicated, was given simultaneously with SSPBI. Patient, tumour and treatment related characteristics are listed in Table 1, respectively. In Table 2, we reported the abbreviations for the polymorphic sites. The genotyping procedure was successful in 57 patients. The observed allele frequencies of the polymorphic genes analyzed were comparable to those reported for European populations in the dbSNP database and are shown in Figure 1.

**Table 1:** Main patient and tumor characteristics

**Age (years)**	median (range)	66 (51-87)
**Tumor stage**	Tis/T1/T2	1/48/8
**Nodal stage**	N0/N1	54/3
**Chemotherapy**	yes/no	15/42
**Hormone-therapy**	yes/no	52/5
**Follow-up (months)**	median (range)	38 (19-50)

**Table 2:** Polymorphism abbreviations

Gene	NCBI ds SNP ID	homozygote wt	heterozygote	Homozygote mut
***XRCC1 G28152A *(Arg399Gln)**	rs25487	GG (399 Arg/Arg)	GA(399Arg/Gln)	AA (399 Gln/Gln)

***XRCC3 C18067T *(Thr241Met)**	rs861539	CC (241Thr/Thr)	CT(241Thr/Met)	TT (241Met/Met)

***XRCC3 A4541G *(5'UTR)**	rs1799794	AA	AG	GG

***GSTP1A313G *(Ile105Val)**	rs1695	AA (105 Ile/Ile)	AG (105 Ile/Val)	GG (105 Val/Val)

***RAD51 G135C *(5'UTR)**	rs1801320	GG	GC	CC

**Abbreviations**: *NCBI *= National Center for Biotechnology Information, *ID *= identification

With a median follow-up 38 months (range: 19-50 months), the G1, G2 and G3 subcutaneous fibrosis, corresponding to a marked increased density and firmness on palpation with/without retraction/fixation, were observed in 23 (40%), 18 (32%) and 7 (12%) patients, respectively. While the G2 and G3 fat necrosis were observed in 1 (2%) and 1 (2%) patient, respectively.

Late moderate-to-severe (≥ G2) subcutaneous fibrosis or fat necrosis were more frequent (64% vs 38%) in patients with the mutation or heterozygote (aa/Aa) genotype of *GSTP1 *(Ile105Val) with greater odds (OR = 2.9; 95% CI, 0.88-10.14, *p *= 0.047 Chi-square test).

No statistical significant increase/decrease of ORs was observed with other SNPs or their combination. In particular, no correlation was found between late toxicity and mut/het *XRCC1 *Arg399Gln, mut/het *XRCC3 *A4541G or mut/het *XRCC3 *Thr241Met or mut/het *RAD51*. Table 3 shows a summary of a statistical analysis.

**Table 3:** ORs of ≥ G2 fibrosis or fat necrosis for different polymorphisms and their combination

Polymorphisms	Genotype	≥ G2 fibrosis or fat necrosis	OR (95% CI)	*p*-value (*)	*p*-value (§)
*XRCC1 *(Arg399Gln)	AA	45%	1		

	aa/Aa	54%	1.41 (0.44-4.58)	0.514	0.599

*XRCC3(*A4541G)	aa/Aa	44%	1		

	AA	53%	1.43 (0.45-4.71)	0.494	0.596

*XRCC3*(C18067T)	AA/Aa	51%	1		

	aa	33%	0.49 (0.04-3.75)	0.413	0.670

*GSTP1*	AA	38%	1		

	aa/Aa	64%	2.9 (0.88-10.14)	**0.047**	0.064

*RAD51*	AA	48%	1		

	aa/Aa	67%	NA #	**0.9751**	0.6115

**Abbreviations**: (*) Chi-squared, (§) Fisher's exact Test, #: not applicable due to the limited number of events

**Figure 2:**
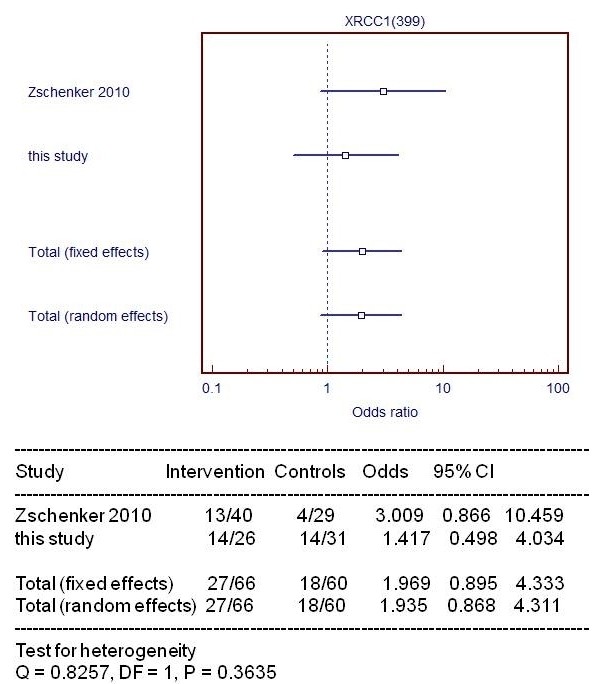
**Forest plot summarizes a pooled analysis of G2 or more fibrosis/fat necrosis distinguishing patients with/without XRCC1 399Gln**. The mutation is toxic or protective when OR is higher or lower than 1, respectively.

**Figure 3:**
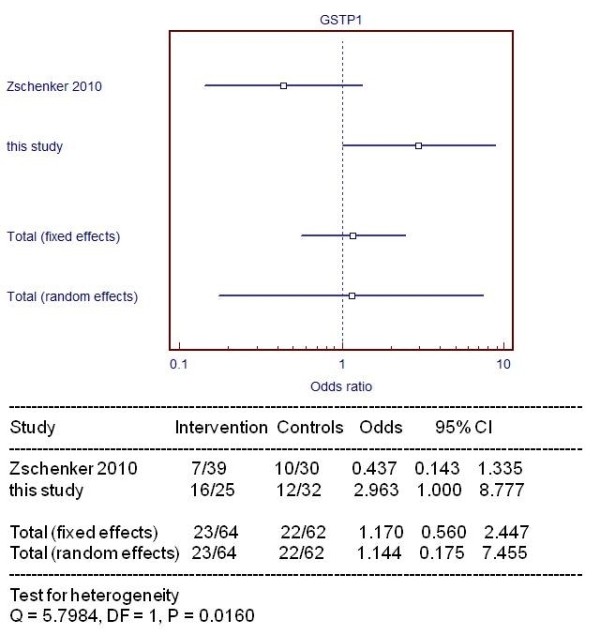
**Forest plot summarizes a pooled analysis of G2 or more fibrosis/fat necrosis distinguishing patients with/without het/mut GSTP1**. The mutation is toxic or protective when OR is higher or lower than 1, respectively.

Forest plots of ≥ G2 fibrosis or fat necrosis in breast cancer patients against het/mut *GSTP1 and XRCC1 *Arg399Gln are shown in Figures 2 and 3, respectively, reporting a meta-analysis of data available from literature.

## Discussion

Recently partial breast irradiation has been proposed in a particular subgroup of patients at low risk of local recurrence. In agreement with this approach, we tested a new schedule at our Institute naming it SSPBI after BCS [8,28]. Due to the major expected killing efficacy of the single dose, unfortunately a incidence of fibrosis/fat necrosis was observed in 44% of our patients.

Generally the moderate-to-severe fibrosis after conventional fractionation is generally observed in 13.5% [35] of patients at 10 years; thus a lot of patients to obverse the same number of complications observed in our cohort (44%). Moreover, the single dose is expected to be more difficult to be repaired, enhancing the scenarios in which the mechanism of protect against *ROS *damage or *DNA repair *fails.

It is for this reason, we focused our attention on SNPs evaluation that may help design a clinical approach and explain basic phenomena such as subcutaneous fibrosis or fat necrosis. Fibrosis is a complex tissue response characterized by a massive deposition of extra cellular matrix (*ECM*) molecules (collagens, non collagenous glycoproteins, glycosaminoglycans, proteoglycans) and excessive fibroblast proliferation. Under oxidative stress generated by ionization radiation, *ROS *levels can increase dramatically, and this may result in significant damage to cell structures. Accordingly, in the cellular compartments, the response to oxidative stress can activate a series of processes including *DNA repair*, antioxidant enzymes, cell cycle arrest and secretion of pro-inflammatory cytokines such as *TNF-α,TGF-β1,IL1, IL6 *and many growth factors in the irradiated tissue. Some authors reported that a coordinated cellular response after radiation occurs, like the involvement antioxidant enzymes (such as superoxide dismutases, catalases, lactoperoxidases, glutathione peroxidases and peroxiredoxins) to protect themselves against *ROS *damage [11-13].

Reduced mechanisms of cell protection resulting from functional polymorphisms in several genes involved in these processes may be associated with the development of late side effects following RT [36]. For these reasons, we decided to investigate genetic variation in enzymes involved in the detoxification process of oxidative stress products, such as *GSTP1*, and its possible correlation with susceptibility to late complications after RT [37,38].

In particular, we found that the polymorphic variant (aa, Aa) of *GSTP1 (Ile105Val)*, producing a protein with reduced activity, is associated with higher risk of developing (G2 or more) a fibrosis or fat necrosis. In fact, Forest plot shows behaviour as toxic agent for GSTP1.

The role of GSTP1 is debated in literature for example Zschenker et al.[39] reported a no statistically significant reduction in G2/G3 fibrosis (like-protective), Kuptsova et al.[40], also in analyzing breast cancer patients found no difference for fibrosis due to the relative small number of patients with this side effect. While Edvardsen et al. [9] reported no association with fibrosis but with an enhanced risk of pleural thickening (like-toxic).

In exacting, *GSTP1 *is involved in the regulation of cell proliferation, apoptosis, stress response, phase II metabolism, oncogenesis, tumour progression and drug resistance. A number of recent studies [11-13] support the role of *GSTP1 *in cell cycle control through the regulation of *c*-*Jun amino-terminal kinase (JNK*) and its indirect role in cellular signalling with interaction with cellular proteins: *TNF-α, TRAF2 cytochine, transcription factor response gene AP-1*.

**Figure 4:**
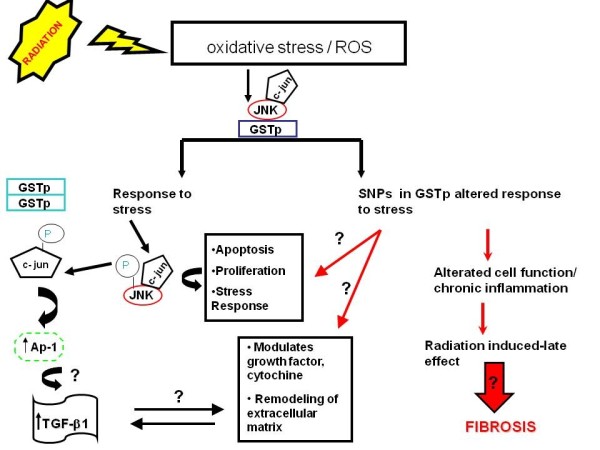
**GSTP1 rule in stress response system**.

As shown in Figure 4, under no stress condition *GSTP1 *interacts with *c-Jun amino-terminal kinases (JNKs) *and represses their activity. After treatment with RT, the concentration of *ROS *in the cell increase and causes the dissociation of *GSTP1-JNK *complex through the oligomerization of *GSTP1 *from monomer to dimer. Subsequently, the released *JNK kinase *recovers its functional activity and can be phosphorylated and phosphorylate *c-jun*. The consequent phosphorylation of *c-jun *activates the transcription of *AP-1 *(stress responsive factor) [41], that is involved in over expression of *TGF-β1 *(*Transforming Growth factor β1) *at sites of RT-induced injury. The critical role of *TGF-β1 *in beginning, expansion and perseverance of fibrosis should be important for preventing/reducing the radiation-induced wound, also including loss of parenchymal cells and excess of fibrous tissue. Furthermore, *TGF-β1 *modulates the activities of cytochine, *TNF-α (Tumour Necrosis Factor alpha), basic fibroblast growth factor (bFGF), granulocyte macrofage colony-stimulating factor (GM-CFS) IL-1, IL-4(interleukins) and connective tissue growth factor (CTGF) *that are deregulated after radiation [42-45]. Thus, this figure suggests that GSTp1 could be indirectly correlated with the regulation of *TGF-β1 *by the *AP-1 *path [46,47].

In accordance with our data, we speculate that the occurrence of fibrosis observed in our cohort of patients may be correlated to the altered regulation of *TGF-β1 *induced by *GSTP1 105Val *polymorphic variant. In this connection, it will be of interest to address this important issue in future studies evaluating the expression levels of *TGF-β*1 in patients bearing *GSTP1 *polymorphism.

In this same cohort of patients, we also evaluated the effects of polymorphism encoding enzymes involved in *DNA repair*, such as *XRCC1 *and *XRCC3*, in relation to fibrosis or fat necrosis. In fact, the cellular pathways of DNA repair more involved in the response to radiation injury are *DSBR *and *BER *In vitro and in vivo studies have shown that polymorphisms of genes involved in these two mechanisms of *DNA repair *may influence the cellular sensitivity to RT [48-50]. Our results showed no significant association between *XRCC3 C18067T *and radio-sensitivity in agreement with studies by Andreassen et al. and Chang-Claude et al. [51-53] in breast cancer patients or by Alsbeish et al. in head and neck cancer patients [54].

An association between wild type *XRCC3 C18067 *and an increased rate of late toxic effects, such as subcutaneous fibrosis, were found in breast cancer [55] and prostate [56].

No statistical significant association between *XRCC1 Arg399Gln *and radio-sensitivity was found in our study, as well as in other studies [17,19,57]. However, Forest plot showed a behaviour as toxic agent of mut/het *XRCC1 Arg399Gln *in agreement with an increased rate of lung effects in non small cell lung cancer patients. [58].

Finally, no correlation was found between late toxicity mut/het *XRCC3 A4541G *and mut/het *RAD51*.

Our low correlation between incidence of G2 or more fibrosis or fat necrosis and alleles/patient is probably due to the low number of patients with G2 or more fibrosis or fat necrosis. Another issue to consider is that in comparison of other findings some differences are expected due to the types of adverse reactions studied, the length of follow-up for observing side effects, as well as, the additional patient-related factors.

## Conclusions

The presence of some SNPs involved in DNA repair or response to oxidative stress seem to be able to predict late toxicity. This study, although affected by a limited number of patients, has a power of the study statistically sufficient to suggest that SNP in GSTP1 gene could be useful to predict late toxicity in BC patients who underwent SSPBI. Further data are needed to confirm these preliminary results. Moreover, future research will focus on the performance of many additional SNPs in other genes that are associated with the development of radiation toxicity.

## Abbreviations

APBI: Accelerated partial breast irradiation; BER: Base excision repair; BC: Breast cancer; BCS: Breast conserving surgery; CEU: Frequencies in European population; CIs: Confidence intervals; CMF: Cyclophosphamide methotrexate 5-fluorouracile; CTCAE: Common terminology criteria for adverse events; dNTP: Deoxynucleotide triphosphate; EC: Epirubicin cyclophosphamide; EDTA: Ethylenediaminetetracetic acid; EIC: Extended intraductal component; FEC: 5-fluorouracile epirubicin cyclophosphamide; GSTs: Glutathione-S-transferases; HR: Homologous recombination; ID: Identification; MVA: Multivariate analysis; NCBI: National Center for Biotechnology Information; ORs: Odds ratios; PCR: Polymerase chain reaction; PBI: Partial breast irradiation; RT: Radiation therapy; rs: Reference sequence; ROS: Reactive oxygen species; SSPBI: Shot partial breast irradiation; SNPs: Single nucleotides polymorphisms; XRCC1: X-ray repair cross-complementation group 1; XRCC3: X-ray repair cross-complementation group 3; WBI: Whole breast irradiation; 3D-CRT: 3D conformal radiation therapy.

## Competing interests

The authors declare that they have no competing interests.

## Authors' contributions

FE, PP, SL conceived the study and obtained grant funding, coordination of the original study, coordinated genotyping efforts, supervised data analysis, and drafted the manuscript. VB, FF and GB participated in data management and statistical analysis, and in drafting the manuscript. GC and LB participated in the design of the original study, data collection and patient management, and in drafting the final manuscript. CG, MP, and BG participated in design of original study, and participated in drafting of final manuscript. All authors read and approved the final manuscript.
